# Identification of Transcription Factor/Gene Axis in Colon Cancer Using a Methylome Approach

**DOI:** 10.3389/fgene.2020.00864

**Published:** 2020-07-31

**Authors:** Jiayu Zhang, Bo Li, Kexin Shen, Huaiyu Zhang, ZiJian Gong, Huaqing Shi, Yang Jiang

**Affiliations:** ^1^Department of Gastrointestinal Colorectal and Anal Surgery, The Third Hospital of Jilin University, Changchun, China; ^2^Department of Gastrointestinal Colorectal and Anal Surgery, China-Japan Union Hospital of Jilin University, Changchun, China; ^3^Department of Gastrointestinal and Colorectal Surgery, China-Japan Union Hospital of Jilin University, Changchun, China; ^4^General Surgery Department, People’s Hospital of Dulbert Mongolian Autonomous County, Daqing, China

**Keywords:** colon cancer, transcription factor, motif, DNA methylation, survival

## Abstract

Colon cancer is one of the most commonly diagnosed cancers worldwide. Both environmental and molecular characters can influence its development. DNA methylation has been heralded as a promising marker for use in cancer prevention, diagnosis, and treatment. It has been shown to facilitate cancer progression through multiple mechanisms. Changes in DNA methylation can inhibit or promote the binding of transcription factors (TFs) and further disturb gene regulation. Detection of DNA methylation-mediated regulatory events in colon cancer are critical for mining novel biomarkers. Here, we explore the influence of CpG sites located at promoter regions of differentially expressed genes and identify methylation–gene relationships using expression–methylation quantitative trait loci. We find that promoter methylation sites mainly negatively regulate the corresponding genes. We also identify candidate TFs that can bind to these sites in a sequence-dependent manner. By integrating transcriptome and methylome profiles, we construct a TF–CpG–gene regulatory network for colon cancer, which is used to determine the roles of TFs and methylation in the transcription process. Finally, based on TF–CpG–gene relationships, we design a framework to evaluate patient prognosis, which shows that one TF–CpG–gene triplet is significantly associated with patient survival rate and represents a potential novel biomarker for use in colon cancer prognosis and treatment.

## Introduction

Colon cancer is one of the leading causes of morbidity and mortality globally (Hobday and Erlichman, 2002). The pathopoiesis of colon cancer is considered to be a polystage and complex process (Rupnarain et al., 2004). Abnormal changes in gene expression have an important role in the development of colon cancer (Sabates-Bellver et al., 2007). The aberrant status of upstream genetic, epigenetic, and transcriptional regulators can contribute to these gene expression changes (Vonlanthen et al., 2014). Previous studies have highlighted and characterized the effects of transcription factors (TFs) in colon cancer (Zhou and Guo, 2018). However, systematic identification of DNA methylation disturbed TFs is still required, and the underlying regulatory mechanisms in colon cancer are poorly characterized.

DNA methylation, one of the best-known epigenetic modifications, has been shown to regulate gene expression in a temporal and spatial specific manner (Schneider et al., 2010; Selva et al., 2017). Alterations to the methylome can promote cancer development via pathway modules (Saghafinia et al., 2018). In colon cancer, hypermethylation of tumor suppressor genes contributes to carcinogenesis through silencing of transcription (Ng and Yu, 2015). Abnormal DNA methylation sites can participate in cross-talk with TFs and affect downstream transcription regulation. Blattler and Farnham (2013) concluded that many TFs bind to unmethylated sites in gene promoter regions. High-throughput technologies have enabled both transcriptome and epigenomic measurements on a genome-wide scale, facilitating the mining of aberrant events in cancer. Thus, detecting activated TFs in colon cancer, combined with DNA methylation status and TF–gene regulatory relationships, will be helpful for colon cancer research.

In this study, we detected differentially expressed genes in colon cancer and focused mainly on determining the upstream regulators of these genes. Based on the relationships between CpG sites and genes, and TF-motif-enriched CpG sites, we constructed a TF–CpG–gene network for colon cancer by integrating transcriptome and methylome profiles. The identified network provides guidance for some dysregulated genes in colon cancer. We also built a comprehensive framework to measure the prognostic efficiency of these TF–CpG–gene relationships. Overall, the findings of this study provide novel guidance for colon cancer research.

## Materials and Methods

### Methylation and Expression Datasets

Processed gene expression profiles and methylation datasets for colon cancer (TCGA-COAD) were downloaded from the UCSC Xena archive^1^. Gene expression values (fragments per kilobase million) were derived from RNA sequencing data and were log_2_ transformed. We only retained samples found in both datasets. In total, there were 306 colon cancer samples and 19 adjacent mucosa tissue samples.

### Identification of Differentially Expressed Genes

We obtained gene annotation files from the GENCODE database (v. 22) (Frankish et al., 2019) and retained the protein-coding genes; we refer these protein-coding genes simply as “genes” in this study. Next, we retained genes that were expressed in at least 50% of samples. Differentially expressed genes were identified based on t-tests and fold change (FC), *p*-values were adjusted using the false discovery rate (FDR) method; genes with *t*-test FDR < 0.05 and log_2_|FC| > 1 were regarded as differentially expressed.

### Methylation–Gene Correlation Analysis

DNA methylation levels of colon samples were measured by 450 K Illumina Infinium HD Methylation Assay (Agha et al., 2019), which can access the methylation status of more than 450,000 CpG sites in the human genome. We first filtered out probes that had single nucleotide polymorphisms located in or close to the probe sequence. The remaining probes were annotated in gene promoter regions (±3 kb from the transcription start site) (Wang et al., 2018) using bedtools (Quinlan, 2014). We tested the correlation of each methylation site–gene pair using Pearson statistics. Significantly correlated pairs (FDR < 0.05) were referred as expression–methylation quantitative trait loci (emQTL).

### Collection of Functional Genes

We collected 167 clinically actional genes from the TARGET database^2^ (Van Allen et al., 2014). We searched the MalaCards database (Rappaport et al., 2014) for colon cancer-related genes using the keyword “colon cancer” and obtained 92 protein-coding genes.

### Motif Enrichment Analysis Within Methylation Sites

We downloaded transcription binding profiles (motifs) from the MEME suite (Bailey et al., 2015). For each promoter methylation site, we used FIMO software (Grant et al., 2011) with default parameters to scan motif occurrence in the 100-bp flanking region each side of the CpG site and retained motifs that covered the corresponding CpG sites. For each such motif, we calculated its odds ratio as well as 95% confidence interval relative to the background CpG sites, according to a previously published method (Yao et al., 2015).

### Construction of TF–Gene Regulatory Relationships

Previous studies have demonstrated that DNA methylation can positively or negatively affect TF-binding events and alter the corresponding gene expression (Zuo et al., 2017), while more recent researches revealed that DNA methylation can be promoted or inhibited by the DNA-binding (TFs) (Blattler and Farnham, 2013; Shakya et al., 2015; Heberle and Bardet, 2019). Therefore, for probes containing at least one enriched motif, we tested the correlation between the corresponding TF expression level and methylation level of this site using Pearson correlation statistics, retaining TF–methylation site pairs with FDR < 0.05. We also identified TFs and CpG-related genes that were significantly correlated (Pearson correlation FDR < 0.05). Thus, we obtained TF–CpG–gene triplets in which each element had significant correlations with the others.

### Prognostic Analysis

We retrieved colon cancer survival data using the TCGAbiolinks package (Colaprico et al., 2016). By applying multivariate Cox proportional regression model, we obtained *p*-values for the TF, CpG site, and gene of each triplet, and generated a combined *p*-value using Fisher’s combination test (Hwang et al., 2005). The TF–CpG–gene triplets that were significantly associated (*p* < 0.05) with survival rate are shown in Table 1.

**TABLE 1:** Significant survival-related TF–CpG–gene relationships based on the multivariate Cox proportional regression model.

**TF**	**CpG site**	**Gene**	**Combined *p*-value**
BATF3	cg00244882	ITIH5	0.016
FEV	cg17978562	EDIL3	0.004
NEUROD1	cg18618334	CXCL12	0.01
GLIS1	cg22444507	ITIH5	0.01

To evaluate the efficiency of the obtained triplets in predicting the survival of patients, 292 patients were randomly divided into training (*n* = 146) and testing (*n* = 146) sets. For each triplet, we evaluated the association between TF, CpG site, gene and survival through multivariate Cox regression analysis in the training set. Next, we performed survival analysis based on the resulting TF–CpG–gene triplets. According to the TF, CpG, and gene coefficients from the Cox regression model, we assigned a risk score to each colon cancer sample as follows:

Risk⁢score=∑α⁢T⁢F+β⁢M⁢e⁢t⁢h+γ⁢G⁢e⁢n⁢e

where α is the coefficient of TF, β is the coefficient of CpG, γ is the coefficient of Gene; and *TF*, *Meth*, and *Gene* represent the corresponding values of the TF, CpG site, and gene in the cancer samples.

We further divided the cancer samples into low- and high-risk groups based on the median risk score across samples and performed Kaplan–Meier estimation between the two groups; *p*-values were calculated by the log-rank test.

### Statistical Analysis

Statistical analysis was performed using R 3.6.1 framework. During the differentially expressed genes identification process, we applied Student’s *t*-test. Besides, *p*-values derived from differential expression analysis, correlation analysis among TFs, DNA methylation and genes were adjusted using the FDR method, FDR < 0.05 was used to filtrate significant results. Kaplan–Meier survival curves were plotted for different groups of patients, the difference between the two groups was calculated by log-rank test.

## Results

### Dysregulated Genes in Colon Cancer

**FIGURE 1:**
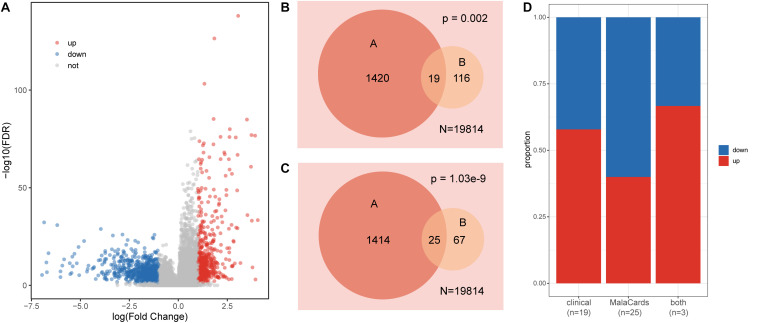
Characteristics of gene expression in colon cancer. **(A)** Volcano plot of dysregulated genes in colon cancer. **(B)** Venn diagram displaying the relationships between dysregulated genes (A set) and clinical action genes (B set); *N* is the total gene number. **(C)** Venn diagram displaying the relationships between dysregulated genes (A set) and colon cancer-related genes (B set); *N* is the total gene number. **(D)** The proportion of up- and downregulated genes in each set.

Genes that display differences in expression distribution between healthy and disease samples are often related to certain diseases or traits. We detected significantly dysregulated genes in the colon cancer dataset using *t*-tests and FC (FDR < 0.05, |log_2_FC| > 1). Among the differentially expressed genes, 623 were upregulated and 816 were downregulated (Figure 1A). We further explored the expression patterns of clinical action genes and colon cancer-related genes. Among these dysregulated genes, 19 were clinical action genes and 25 were related to colon cancer, according to the TARGET and MalaCards databases, respectively. Both the clinical action genes and colon cancer-related genes tended to display a differential expression pattern (hypergeometric test *p* < 0.01, Figures 1B,C), and there was no obvious difference between the up- and downregulated genes in these functional gene sets (Fisher’s exact test, *p* = 0.73, Figure 1D).

### Identification of Correlated Methylation–Gene Pairs as emQTLs

**FIGURE 2:**
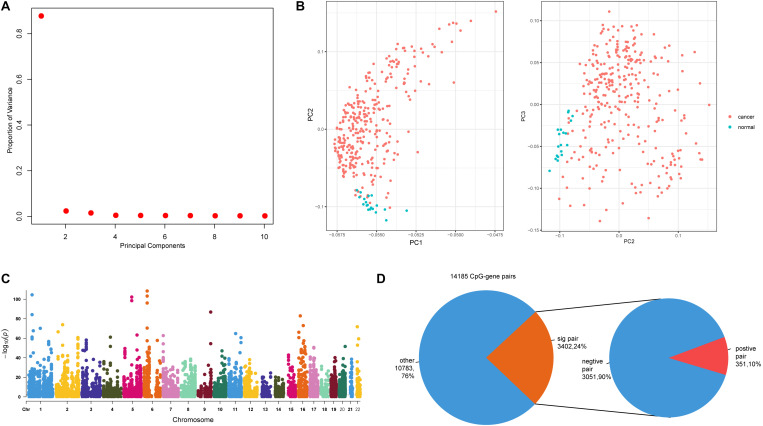
Relationships between promoter CpG sites and host genes. **(A)** The proportion of variance of the first three principal components (PCs). **(B)** Scatter plot of PC1 and PC2, and PC2 and PC3. **(C)** Manhattan plot of genome-wide *p*-values of CpG–gene pairs; dashed line indicates *p* = 0.01. **(D)** The first pie plot shows the proportions of significant and non-significant CpG–gene pairs. The second pie plot shows the positive and negative pairs derived from the significant pairs.

Next, we sought to explore the relationships between methylation sites and dysregulated genes. For the 1,439 dysregulated genes, there were 14,066 CpG sites mapping to 1,314 genes. We next performed principal component analysis (PCA) using these 14,066 CpG sites, and further examined the characteristics of colon cancer and adjacent mucosa samples based on the first three principal components (Figure 2A). Colon cancer and adjacent mucosa samples have distinct methylation character based on PCA result (Figure 2B). This suggests that DNA methylations within the dysregulated gene promoters have different patterns in cancer samples compared with adjacent mucosa samples. Previous studies have highlighted the role of DNA methylation in gene regulation during cancer progression (Baylin and Jones, 2011). We identified methylation–gene pairs (Figure 2C) using correlation statistics and denoted the significantly correlated pairs as emQTLs (Fleischer et al., 2017). Among the 14,185 methylation–gene pairs, nearly 24% (3,402) pairs had significant relationships (Figure 2D). Furthermore, most (90%) emQTLs showed negative regulation, which is consistent with previous findings that DNA methylation can block promoter activity and repress gene expression (Jain, 2003).

### Characterization of DNA Methylation-Mediated TF-Gene Axis in Colon Cancer

**FIGURE 3:**
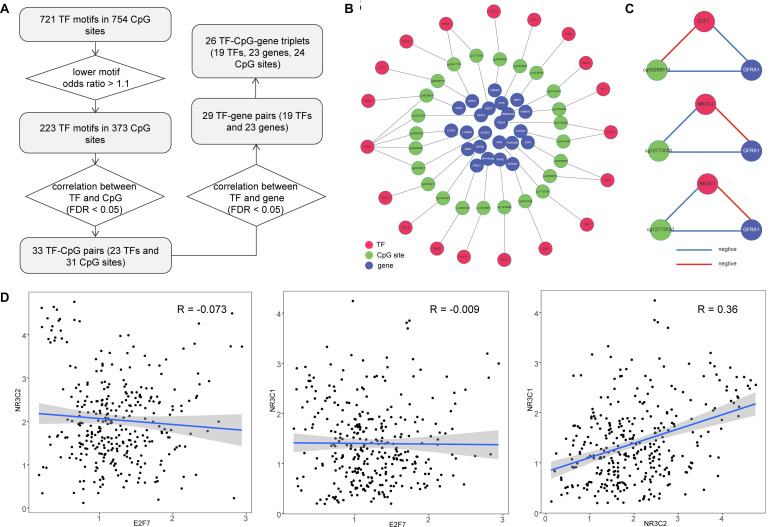
Construction of TF–CpG–gene network for colon cancer. **(A)** Workflow for identifying TF–CpG–gene relationships. **(B)** The network of correlated TF–CpG–gene triplets. **(C)** Correlation orientation among TF–CpG–gene triplets. **(D)** Scatter plots show the correlation among E2F7, NR3C2, and NR3C1.

DNA methylation sites have been shown to play an important part in regulating TF binding events (Yao et al., 2015). We explored the occurrence of TF motifs within emQTLs. During this process, we required CpG sites to be located within the motif region and identified 721 TF motifs in 754 CpG sites. We also expect TF motifs would be more likely to bind emQTLs compared with all CpG sites in gene promoters, so we calculated odds ratios for these motifs and identified 223 TF motifs (lower odds ratio >1.1) in 373 CpG sites. The relationship between each TF and the corresponding CpG site was determined by Pearson correlation (FDR < 0.05); in this way, we obtained 33 TF–CpG pairs, comprising 23 TFs and 31 CpG sites. Furthermore, we obtained the correlation relationships for TF–gene pairs (Pearson correlation FDR < 0.05) and identified 29 TF–gene pairs (Figure 3A). In total, we obtained 26 TF–CpG–triplets, comprising 19 TFs, 24 CpG sites, and 23 genes. Finally, we constructed a network based on the relationships among the identified TFs, CpG sites, and genes (Figure 3B). Network results showed there were two CpG sites (cg06298519, cg25617725) within the GFRA1 promoter, cg06298519 was associated with TF E2F7, while cg25617725 was associated with TF NR3C2 and NR3C1, respectively (Figure 3B). The methylation level of cg06298519 and cg25617725 were negatively correlated the expression of GFRA1. TF NR3C2 and NR3C1 all showed positive correlation with GFRA1, whereas E2F7 was negatively correlated with GFRA1 (Figure 3C). This indicates NR3C2 and NR3C1 potentially cooperate with each other to regulate GFRA1. Next, we tested the expression correlation among E2F7, NR3C2, and NR3C1 (Pearson correlation test). E2F7 was negatively correlated with NR3C2 and NR3C1, respectively (Figure 3D). Therefore, although TF E2F7 display opposite regulation effect on GFRA1 when comparing with NR3C2 and NR3C1, it did not have a competitive role with them in colon cancer. Dysregulated genes in this network may be caused by cooperation between DNA methylation sites and TF binding in their promoters; thus, determining their combined roles in cancer samples will be beneficial for colon cancer treatment and prognosis.

### Dissection of Prognostic Efficiency of TF–CpG–Gene Triplets in Colon Cancer

**FIGURE 4:**
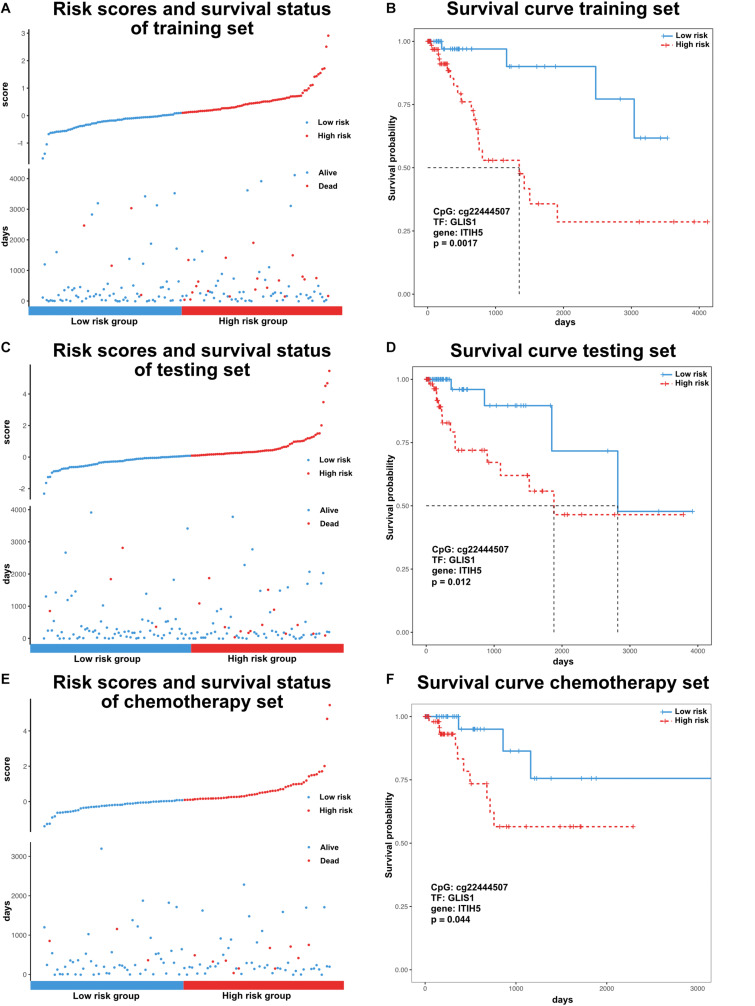
Survival analysis of colon cancer samples based on the risk model. **(A)** Ranked risk scores across colon cancer samples of the training set (top). Distribution of survival status in colon cancer samples of the training set (bottom). **(B)** Kaplan–Meier curve for two risk groups of the training set. **(C)** Ranked risk scores across colon cancer samples of the testing set (top). Distribution of survival status in colon cancer samples of the testing set (bottom). **(D)** Kaplan–Meier curve for two risk groups of the testing set. **(E)** Ranked risk scores across colon cancer samples of the chemotherapy set (top). Distribution of survival status in colon cancer samples of the chemotherapy set (bottom). **(F)** Kaplan–Meier curve for two risk groups of chemotherapy set.

Previous research has identified many prognostic markers for cancer, including DNA methylation sites and coding genes (Cheng et al., 2009; Hao et al., 2017). However, the majority of these studies were limited to one type of molecular level. In order to evaluate the prognosis of patients in a more comprehensive way, we analyzed the effects of DNA methylation sites, TFs, and genes from our dissected triplets on patient survival time. For each triplet, we used a training set to construct a risk model (methods, α = 4.18, β = 0.61, γ = −0.80) based on the risk coefficients by applying a multivariate Cox proportional regression model. In the training set, the GLIS1_cg22444507_ITIH5 triplet was significantly associated with survival time (*p* < 0.05, Figures 4A,B). Similarly, in the testing set, patients were assigned risk value using the same model as the training set and can also be significantly divided into low- and high-risk groups (*p* < 0.05, Figures 4C,D). Furthermore, consider the treatment effect on survival, we tested the efficiency of GLIS1_cg22444507_ITIH5 triplet on a subset of patients that only received chemotherapy (*n* = 152). The result showed GLIS1_cg22444507_ITIH5 was associated with the survival rate for patients that only received chemotherapy (*p* < 0.05, Figures 4E,F). These results suggest the GLIS1_cg22444507_ITIH5 triplet is the potential biomarker for colon cancer prognosis.

## Discussion

In the present study, we identified 1,439 significantly differentially expressed genes between colon cancer and adjacent mucosa tissue samples. Of these genes, 19 were identified as clinical action genes and 25 were shown to be associated with the development of colon cancer. Differentially expressed genes may have important roles in tumor progression, diagnosis, and prognosis (Liang and Pardee, 2003). Mining crucial markers among these genes and investigating their upstream regulators will be beneficial for cancer treatment. We mapped CpG sites into gene promoters and identified emQTLs to further elucidate the regulatory roles of the corresponding CpG sites. We found that most of the obtained emQTLs had negative relationships, indicating that promoter methylation sites mainly repress gene regulation in colon cancer (Curradi et al., 2002).

DNA methylation of regulatory elements can modulate TF binding to DNA (Heberle and Bardet, 2019). Detection of TF–methylation binding events can provide information about the origin of gene dysregulation; thus, we focused on the emQTLs and further mining of TF binding events. To analyze the occurrence of TF-related motifs in each selected CpG site, we examined the enrichment status of each motif. As expected, the surrounding sequences of the CpG sites had enriched motifs, demonstrating that these sites could bind TFs. We further constructed a TF–CpG–gene network for colon cancer using transcriptome and methylome datasets. These triplets represent potential biomarkers for colon cancer and may have applications in novel treatment strategies.

**FIGURE 5:**
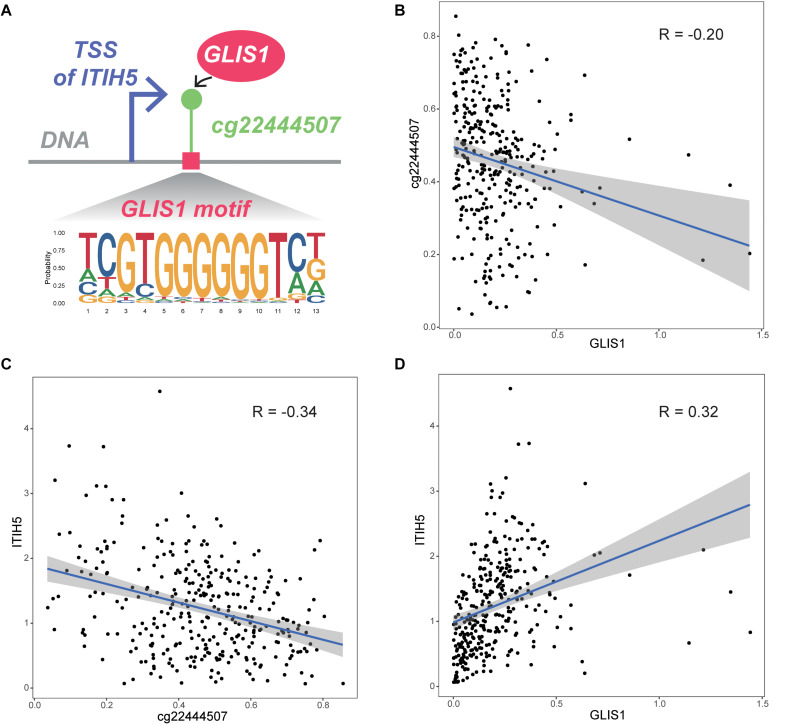
Biological interpretation of the GLIS1_cg22444507_ITIH5 triplet. **(A)** The biological mechanism of the GLIS1_cg22444507_ITIH5 triplet. **(B)** Scatter plot of expression level of GLIS1 and DNA methylation level of cg22444507 across samples. **(C)** Scatter plot of DNA methylation of cg22444507 and expression level of ITIH5 across samples. **(D)** Scatter plot of expression level of GLIS1 and ITIH5 across samples.

We initially obtained four TF–CpG–gene triplets that were significantly associated with patient survival time. Based on the resulting TF–CpG–gene relationships, we designed a framework to evaluate the prognostic risk score for colon cancer samples. One TF–CpG–gene triplet, GLIS1_cg22444507_ITIH5, could successfully divide colon cancer samples into low- and high-risk groups. Of this triplet, GLIS1 potentially recognize the corresponding motif and bind the DNA sequence around cg22444507 (Figure 5A). GLIS1 binding event may lead to the decreased DNA methylation level of cg22444507 (Figure 5B). Besides, DNA methylation of cg22444507 could negatively regulate the expression of ITIH5 (Figure 5C). Whereas GLIS1 could promote the expression of ITIH5 (Figure 5D). Therefore, GLIS1 and cg22444507 potentially cooperate with each other and affect the expression of ITIH5. ITIH5 (inter-α-trypsin inhibitor heavy chain 5) has been identified as a novel prognostic marker for breast cancer, mediated by promoter hypermethylation (Veeck et al., 2008), and is a novel candidate tumor suppressor gene in colon cancer (Kloten et al., 2014).

However, due to data limitations, we could not validate the prognostic efficiency of GLIS1_cg22444507_ITIH5 in external datasets. We collected data from publicly available Gene Expression Omnibus (GEO) database, used GLIS1_ITIH5 to test the effect on survival without DNA methylation data and found GLIS1_ITIH5 cannot significantly divided patients into low- and high-risk group (*p* = 0.60 in GSE39582, *p* = 0.49 in GSE17536). This indicates the importance of combining TF, DNA methylation and gene in survival prediction for colon cancer. With the generation of the associated data, we will verify the role of GLIS1_cg22444507_ITIH5 in colon cancer. Overall, our study demonstrated the role of cooperation between TFs and DNA methylation in gene regulation in colon cancer, and identified TF–CpG–gene events that may provide guidance for colon cancer prognosis and treatment. In further work, we will continue to study the correlation between TF-CpG-gene network and colon cancer on a deeper level.

## Data Availability Statement

The datasets generated for this study can be found in UCSC Xena archive, https://xena.ucsc.edu/, and The Cancer Genome Atlas (TCGA), https://cancergenome.nih.gov/.

## Author Contributions

YJ and JZ conceived and designed the experiments, and wrote the manuscript. JZ conducted most of the experiments. BL performed some experiments. KS, HZ, ZG, and HS gave advice and supervised some experiments. All authors read and approved the final manuscript.

## Conflict of Interest

The authors declare that the research was conducted in the absence of any commercial or financial relationships that could be construed as a potential conflict of interest.

## Footnotes

1 https://xena.ucsc.edu/

2 https://software.broadinstitute.org/cancer/cga/target
